# Dr. Julius Wenz

**Published:** 1912-06

**Authors:** 


Dr. Julius Wenz of Lancaster, N. Y., died April 25, 1912, aged 68. He was a graduate of Buffalo, 1865.

